# Toxic epidermal necrolysis associated with severe cytomegalovirus infection in a patient on regular hemodialysis

**DOI:** 10.4084/MJHID.2011.004

**Published:** 2011-01-14

**Authors:** Dina Khalaf, Bassem Toema, Nidal Dabbour, Fathi Jehani

**Affiliations:** 1 MSc in Internal Medicine, Cairo University, Egypt; 2 MSc in Experimental Therapeutics, University of Oxford, United Kingdom; 3 Clinical Fellow in Dermatopathology, The State University of New York, USA; 4 MBChB, DIM, MSc, MRCP(UK), FRCPath, Consultant Hematologist

## Abstract

Primary illness with cytomegalovirus leads to latent infection with possible reactivations especially in the immunocompromised patients. Toxic epidermal necrolysis is an immune mediated cytotoxic reaction.

A fifty years old female diabetic hypertensive patient with end stage renal disease was admitted with fever of unknown origin, constitutional symptoms, vague upper gastrointestinal symptoms and skin rash. Upper gastrointestinal endoscopic biopsy confirmed her diagnosis with cytomegalovirus esophagitis and duodenitis. Cytomegalovirus immunoglobulin M and immunoglobulin G levels were negative but polymerase chain reaction showed fulminant viremia. Biopsy of the skin rash was consistent with toxic epidermal necrolysis. Despite treatment with Ganciclovir, intravenous immunoglobulins, and granulocyte colony stimulating factor the patient’s condition rapidly deteriorated and she died due to multiorgan failure, disseminated intravascular coagulopathy and overwhelming sepsis.

Probably there is a true association linking toxic epidermal necrolysis to fulminant reactivation of cytomegalovirus. The aim of this anecdote is reporting a newly recognized presentation of cytomegalovirus.

## Introduction

Primary illness with cytomegalovirus (CMV) leads to latent infection with possible reactivations especially in the immunocompromised patients. Both the primary illness and the reactivations are active CMV infections with viral replication.^1^

Toxic epidermal necrolysis (TEN) is an immune mediated cytotoxic destruction of keratinocytes that express foreign antigens. Most commonly it is drug induced but it may occur secondary to infections, malignancies, and vaccinations. It mimics type IV hypersensitivity reaction with characteristic delayed reaction to an initial exposure and an increasingly rapid reaction with repeated exposure.^2^ The estimated annual incidence of TEN is reported to be between 0.4 and 1.3 cases per million per year and may occur in all age groups. Reported mortality varies from 30 to 50% with the primary cause of death being infection and multiorgan failure.^3^

The incidence of TEN increased to a thousand fold in patients with Human Immunodeficiency Virus and Acquired Immunodeficiency Syndrome.^4^ This is due to an imbalance in the inherent activation and detoxification mechanisms as well as an altered innate immune response. Specific viral infections had been shown to increase CD95 (Fas) and/or Fas Ligand expression and increased sensitivity to Fas/Fas Ligand dependent apoptosis.^5^ Authors have hypothesized that reactivation of human herpesvirus type 6 may seriously interact with some of the enzymes that detoxify the drugs, such as cytochrome P450. The toxic and immunogenic metabolites of these drugs are deposited in the epidermis leading to a series of immune reactions causing TEN.^6^

## Case report

**Figure 1:**
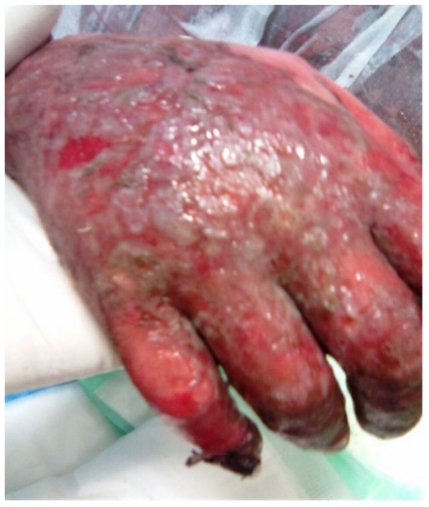
Illustration shows erythroderma and scaly skin of the upper extremity.

**Figure 2:**
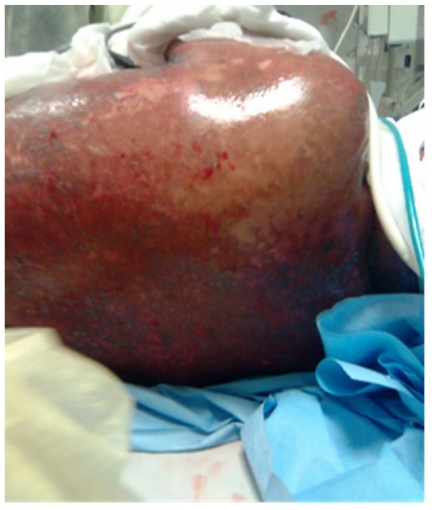
Illustration shows erythroderma and scaly skin of the trunk.

A fifty years old caucasian female patient with positive family history for hypertension and negative family history for malignancy, having hypertension controlled by lisinopril, amlodipine and bisoprolol fumarate, Diabetes mellitus type II (DM II) controlled by short acting regular insulin, and end stage renal disease (ESRD) on regular hemodialysis. She was admitted to the intensive care unit (ICU) with fever of unknown origin (FUO) of fourteen days duration associated with agitation, irritability, tachycardia (120 beats/minute), generalized weakness, anorexia, nausea, vomiting, diarrhea, scratch marks and maculopapular rash (Figures 1 and 2). Sepsis workup was done followed by infusion of empirical intravenous broad spectrum antibiotics with the dose adjusted according to renal function and systemic steroids were started with methyl prednisolone 40 milligrams intravenous infusion once daily.

On Day two, the patient developed severe upper epigastric pain. Upper gastrointestinal endoscopic biopsy confirmed her diagnosis with severe CMV esophagitis and duodenitis. Treatment was started with intravenous Ganciclovir at a dose of 1.25 milligrams/kilogram administered three times/week following each hemodialysis session.

**Figure 3:**
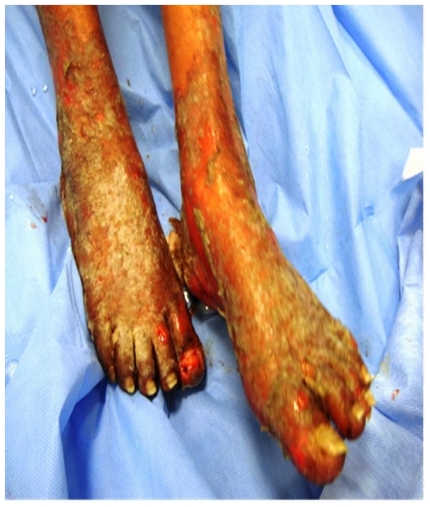
Illustration shows bullous lesions of the lower extremities associated with skin peeling, bleeding and positive Nikolsky’s sign.

**Figure 4:**
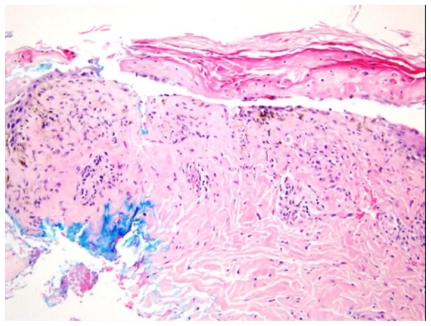
Histopathological examination of the skin biopsy. The black arrows illustrate the pan epidermal necrosis and the red arrows demonstrate the separation of the epidermis from the dermis

On day three the maculopapular rash progressed to erythroderma, followed by development of bullous lesions all over the body associated with skin peeling, bleeding, positive Nikolsky’s sign and mucous membrane involvement (Figures 3). Skin biopsy was done and the pathology showed extensive epidermal necrosis, focal subepidermal necrotic blisters and extensive vacuolar degeneration of dermoepidermal junction with separation of the epidermis from the dermis. The dermis showed melanin incontinence and moderate perivascular lymphocytic infiltrate in the absence of eosinophils, neutrophils and viral inclusions (Figure 4). TEN was confirmed. All the immunoflourescence markers that were done on the skin biopsy showed negative staining with nonspecific granular deposition in the necrotic epidermis. The immunoflourescence markers included Immunoglobulin G (IgG), Immunoglobulin A (IgA), Immunoglobulin M (IgM) and Complement factor 3. On day 15, she developed pneumonia which was complicated by respiratory failure. Intubation and mechanical ventilation were initiated.

On day 48 the patient, whose SCORTEN (severity-of-illness score) was five and expected mortality rate was 90%, passed away due to overwhelming sepsis, shock and multiorgan failure.

## Discussion

The case presented showed suggestive evidence linking CMV to TEN. To associate CMV with TEN, we had to differentiate TEN from similar skin diseases, explore other possible causes of TEN and reactivation of CMV, assess the temporal relationship and biological plausibility, show recognized association with the herpes viruses group and identify CMV.

1. *Differentiate TEN from similar skin diseases:* Differential diagnosis included Staphylococcal Scalded Skin Syndrome (SSSS), Toxic Shock Syndrome, Pemphigus Vulgaris, Bullous Pemphegoid and Bullous Dermatosis of hemodialysis. Skin biopsy in SSSS and Toxic Shock Syndrome shows superficial intra epidermal bullous formation but in TEN there is pan-epidermal necrosis with subepidermal bullous formation. Treatment of SSSS and Toxic Shock Syndrome includes antibiotics, but in TEN the antibiotics may cause the disease.^7^ Pemphigus Vulgaris, Bullous Pemphegoid and Bullous Dermatosis of hemodialysis are usually self limited and occur on the photo-exposed parts but TEN is a rare, potentially life-threatening medical emergency characterized by wide-spread epidermal sloughing of skin accompanied by mucus membrane involvement.^8^
2. *Exploring other possible causes of TEN and reactivation of CMV:* Etiology of TEN includes drugs, malignancies and infections especially in immunocompromised patients with multiple comorbidities. Apart from her regular medications, there was no history of recent new drug or herbal ingestion. Her regular medications (lisinopril, amlodipine, bisoprolol fumarate and short acting regular insulin) are not reported to cause Stevens-Johnson syndrome or TEN. Furthermore, the patient had used her regular medications for many years without developing this severe skin disease. The patient had negative history of malignancy. Multiple chronic diseases and comorbidities as long standing DM II, hypertension, and ESRD, in addition to regular hemodialysis, systemic steroids, prolonged ICU stay and sepsis kept her in an immunocompromised state which increased the risk for reactivation of CMV, augmentation of the viral load and the virulence of CMV, and development of TEN.
3. *Temporal relationship and biological plausibility:* In TEN the clinical features typically include a prodrome of 2 to 3 days characterized by fever, cough, sore throat, and general malaise before the cutaneous manifestations of TEN become apparent. Symptoms of CMV infection in immunocompromised patients cover a broad range, from direct manifestations of viral replication like fever, leucopenia, thrombocytopenia, hepatitis, enteritis, and pneumonia to indirect sequelae like an impaired cellular immune response. It is possible that the fourteen days FUO that the patient experienced before ICU admission represent reactivation of CMV with viral replication that predisposed to TEN when the patient was admitted to ICU and started receiving empirical intravenous antibiotics.^9^
4. *Recognized association with the herpes viruses group:* Cytomegalovirus (human herpesvirus type 5) is a herpes viral genus of the herpesviruses group. High rate of skin reactions to ampicillin (80% to 100%) was noticed in patients with acute Epstein-Barr virus (human herpesvirus type 4). Reactivation of human herpesvirus type 6 has been reported in drug induced hypersensitivity syndrome.^10^
5. *Identification of CMV:* Despite negative CMV IgG level of 15 grams/liter (reference range: 7–16) and IgM level of 0.531 grams/liter (reference range: 0.4–2.3), yet CMV polymerase chain reaction (PCR) was conclusive of fulminant viremia at 680,876 copies/milliliter (Threshold: 350).

## Conclusions

Patients involved are only one (single case report). Cause implicated is CMV. Probably there is a true association linking CMV to TEN. Suggestive reasons are the temporal relationship and biological plausibility, recognition of the association with the herpes viruses group, identification of CMV and all other possible causes of TEN were ruled out.

The hypothesis generated is whether TEN is linked to fulminant CMV infection or not and does CMV trigger an interaction between cytotoxic T-lymphocytes, natural killer cells and keratinocytes or not. Further observational studies are warranted.

Implications for clinical practice include:

- Screening for CMV with PCR as a causative agent for TEN even in the absence of a clear history of offending drug ingestion and even if CMV IgM and IgG levels are negative.
- Systemic steroids are absolutely contraindicated in the treatment of TEN in case the causative agent is fulminant viral infection.

Treatment strategies for TEN associated with CMV include treatment of the cause with Ganciclovir, avoidance of possible offending drugs associated with TEN and avoidance of systemic steroids assuming that most likely the underlying mechanism is interaction of CMV with some of the enzymes that detoxify the offending drugs associated with TEN causing the deposition of the toxic and immunogenic metabolites of these drugs in the epidermis leading to a series of immune reactions that end in TEN.

## References

[1] Ljungman P, Griffiths P, Paya C (2002). Definitions of cytomegalovirus infection and disease in transplant recipients. Clin Infect Dis.

[2] Paul C, Wolkenstein P, Adle H, Wechsler J, Garchon HJ, Revuz J, Roujeau JC (1996). Apoptosis as a mechanism of keratinocyte death in toxic epidermal necrolysis. Br J Dermatol.

[3] Fritsch PO, Sidoroff A (2000). Drug-induced Stevens-Johnson syndrome/toxic epidermal necrolysis. Am J Clin Dermatol.

[4] Rzany B, Mockenhaupt M, Stocker U, Hamouda O, Schöpf E (1993). Incidence of Stevens-Johnson syndrome and toxic epidermal necrolysis in patients with the acquired immunodeficiency syndrome in Germany. Arch Dermatol.

[5] Teraki Y, Shiohara T (1999). Apoptosis and the skin. Eur J Dermatol.

[6] Suzuki Y, Inagi R, Aono T, Yamanishi K, Shiohara T (1998). Human herpesvirus 6 infection as a risk factor for the development of severe drug-induced hypersensitivity syndrome. Arch Dermatol.

[7] Amon RB, Dimond RL (1975). Toxic epidermal necrolysis. Rapid differentiation between staphylococcal- and drug-induced disease. Arch Dermatol.

[8] Ruiz-Maldonado R (1985). Acute disseminated epidermal necrosis types 1, 2 and 3: a study of sixty cases. J Am Acad Dermatol.

[9] Fishman JA, Rubin RH (1998). Infection in organ-transplant recipients. N Engl J Med.

[10] Pullen H, Wright N, Murdoch JMC (1967). Hypersensitivity reactions to antibacterial drugs in infectious mononucleosis. Lancet.

